# Effects and mechanisms of lifespan extension and healthspan promotion induced by *Limosilactobacillus reuteri* (*Lactobacillus reuteri*) A21041

**DOI:** 10.3389/fnut.2026.1852156

**Published:** 2026-07-10

**Authors:** Lili Meng, Lianfei Huang, Enqiu Lu, Zhihui Chai, Zhilong Lu, Shifu Pang, Xinli Wei, Manping Chen, Guilong Xiao, Yanjin Lin, Weifei Luo

**Affiliations:** 1 AIage Longevity Science Corporation Ltd., Shenzhen, Guangdong, China; 2 Guangxi Key Laboratory of Longevity Science and Technology, Guangxi AIage Longevity Science Corporation Ltd., Nanning, Guangxi, China; 3 National Key Laboratory of Non-food Biomass Energy Technology, Institute of Biological Science and Technology, Guangxi Academy of Sciences, Nanning, Guangxi, China; 4 Sichuan AIage Longevity Science Corporation Ltd., Dazhou, Sichuan, China

**Keywords:** *Caenorhabditis elegans*, *Limosilactobacillus. reuteri*, longevity, probiotics, stress resistance

## Abstract

**Background:**

Nowadays probiotics have been widely used in the fields of food, medicine and agriculture. In this study, we investigated the anti-aging effects of the probiotic strain of *Limosilactobacillus reuteri* A21041 using *Caenorhabditis elegans*.

**Methods:**

In this study, lifespan assays, lipid storage experiments, motility tests and stress resistance assays were conducted using *C. elegans* to present the differences between A21041 and control group *Escherichia coli* OP50. Besides, transcriptome sequencing and non-targeted metabolomics analysis were performed to further reveal the mechanism of A21041-mediated lifespan prolongation.

**Results:**

We found that A21041 extended the lifespan of nematodes by 19.27% compared with the control group, and reduced lipid accumulation of *C. elegans* by 54.47% in day 5 and 68.77% in day 7 via lysosome signaling pathway. In addition, A21041 increased the viability of worms under thermal stimulus through up-regulation of *hsp-16.2* and *hsp-110* mRNA levels. Meanwhile, A21041 feeding activated superoxide dismutase (SOD) and enhanced antioxidative ability of nematodes. Of impoatance, A21041 did not extend the lifespan of loss-in function mutants of *daf-2*, *akt-2* and *aak-2*, which suggested that insulin/IGF-1 signaling (IIS) were required for the life-extending effect of A21041. RNA-seq analysis was consistent with our findings above. Gene *gst-4*, which has been confirmed to be highly associated with antioxidant activity and lifespan extension was significantly up-regulated. And RNA sequencing still revealed that ATF-4/CTH-2/H2S pathway might be related to A21041-mediated longevity. Metabolites analysis presented that many metabolites produced by A21041 could protect worms from various damages such as oxidants, inflammatory factors and etc.

**Conclusion:**

In conclusion, our results demonstrated that *L. reuteri* A21041 was able to prolong the lifespan and promote the healthspan of *Caenorhabditis elegans*.

## Introduction

Although lifespan prolongation and anti-aging research are frequent topics of research, their underlying mechanisms remain difficult to study due to the fact that aging and longevity are complex life-long processes defined by genetic, epigenetic, and environmental factors (1). Interestingly, centenarians represent excellent models to explore the relationships between longevity and genetic variation, dietary habits, metabolism, and gut microbiome. Recent studies have shown that the gut microbiome of centenarians harbors unique features that may help centenarians to evade and survive several age-related diseases (2, 3); Pang et al. (2) found that the gut microbiota of centenarians showed a trend toward a more youthful composition. Identification and characterization of specific strains with lifespan extension and anti-aging effects from healthy centenarians are interesting in the field. Thus, efforts to investigate the relationship between the gut microbiome of centenarians and healthy longevity has long been an active area of anti-aging research.

The gut microbiome is an integral part of the human body and plays an essential role in many physiological activities such as immune response and the metabolic process (4). Many beneficial probiotic strains isolated from the human gut belong to *Lactobacillus* and *Bifidobacterium* genus and were wildly applied in food industry, fermentation products, and medicine. Nishida et al. (5) found that long-term use of *Lactobacillus gasseri* CP2305 tablets could improve the mental state, sleep quality, and intestinal microbiota balance of human beings when they were stressed. *Lactobacillus rhamnosus* GG is a widely used probiotic on account of its excellent ability to prevent diarrhea, stimulate the immune response, and even help to prevent some allergic symptoms (6). *Bifidobacterium animalis* subsp. *lactis* HN019 is also a multifunctional probiotic that can ameliorate chronic idiopathic constipation and prevent oral pathogens (7, 8). Fang et al. (9) found that *Bifidobacterium longum* CCFM1029 could alleviate atopic dermatitis by mediating tryptophan metabolism.

The investigation of the effect of probiotic strains on lifespan extension and anti-aging has been expedited by the introduction of *C. elegans* due to its short life and reproduction cycle, low cost, and easy maintenance, as well as a well-characterized genome sequence (10, 11). Importantly, many signaling pathways associated with aging are highly conserved between mammals and *C. elegans*, such as the insulin/IGF-1 signaling (IIS) pathway, mitogen-activated protein kinase pathway (MAPK), and target of rapamycin (TOR) signaling (12, 13). Thus, researchers widely use *C. elegans* to study metabolic diseases, the aging process, microbe-host interactions, kidney diseases, and neurodegenerative diseases such as Alzheimer’s disease and Parkinson’s disease. (14–18). For example, Jones et al. (19) studied lipid metabolism to better understand the basic biology of obesity through a *C. elegans* model; worms harboring the *daf-2* mutation live twice as long as the wild-type strain due to the activity of the DAF-16 protein, the mammalian ortholog of which is FOXO (20, 21). Wang et al. (22) used *C. elegans* as a model animal to test whether blueberry extract can promote longevity and stress tolerance.

Our previous study demonstrated that *L. reuteri* A21041 has an excellent tolerance to artificial gastrointestinal fluid and bile salts and displays a significant anti-inflammatory effect in mouse models (23, 24). Using licensed patents, we have also completed the safety evaluation of this strain which confirmed that this strain was safe. In order to explore the functions of this probiotic more broadly, we conducted a large number of screening experiments. In this study, *C. elegans* was employed to explore the effects of fat reduction and stress resistance of *L. reuteri* A21041 and investigate the underlying mechanism.

## Materials and methods

### Maintenance of bacterial and *Caenorhabditis elegans* strains

*L. reuteri* strain A21041 was isolated from the feces of a healthy centenarian from Beihai City of the Guangxi Zhuang Autonomous Region of China with written consent and the strain was grown at 37 °C using De Man, Rogosa, and Sharpe (MRS; Hopebio, Qingdao, China) broth. *Escherichia coli* OP50 was provided by the Guangxi Academy of Sciences and grown in Luria-Bertani (LB) broth (tryptone, 10 g/L; yeast extract, 5 g/L; NaCl, 10 g/L) at 37 °C overnight.

The *C. elegans* wild type strain N2 was provided by Professor Bing Wang of Guangxi Academy of Sciences and Professor Yanxun Yu from Wuhan University kindly provided the loss-in function mutants of *daf-2* (*e1370*), *akt-2* (*ow393*), *hsf-1* (*sy441*), *pmk-1* (*km25*), *sek-1*(*km4*), *nsy-1* (*ag3*), *aak-2* (*ok524*), *eat-2* (*ad1116*), *skn-1* (*mg570*), *age-1* (*hx546*), *daf-16* (*mgDf47*), *daf-18* (*e1375*), and *daf-28* (*em2980*). All *C. elegans* strains were routinely cultivated at 20 °C on Nematode Growth Medium (NGM) plates, which maintained appropriate living OP50 cells. Before every experiment, *C. elegans* were age-synchronized according to Nakagawa’s study (25), and then the worms at the L4/young adult stage were prepared for experiments.

### Longevity assays in *Caenorhabditis elegans*

Longevity assays were based on Hu’s et al. (26) study, with small modifications. About 200 L4 stage worms were transferred to fresh NGM plates seeded with live *E. coli* OP50 or live *L. reuteri* A21041 at 20 °C (50 worms per plate). Thereafter, worms were transferred every other day until all worms were dead. A worm that did not respond to mechanical stimulation was scored as dead. The time when the L4 stage nematodes were picked onto the plates was recorded as day 0. Three independent longevity assays were performed.

### Body size and brood size assays

For body size tests, about 100 worms were transferred to fresh NGM plates seeded with *E. coli* OP50 or A21041 at 20 °C. For each group, 20 worms were used for body size photographs on day 1, day 3, and day 5. Images of worms were obtained using a Leica Microsystems CMS GmbH microscope (Leica, Shanghai, China) and their body area and length were analyzed using ImageJ software. For brood size assays, one L4 worm was transferred to one NGM plate seeded with *E. coli* OP50 or A21041 at 20 °C, and the eggs of each worm was calculated every day until there were no eggs on the plate, using at least 10 worms for each group. Three independent body and brood size assays were performed.

### Motility assays

Locomotive function is important, especially for aged nematodes (27). Motility assays were determined by head swing rate and body bending rate to evaluate the effects of worms fed with *E. coli* OP50 or *L. reuteri* A21041. L4 stage worms were transferred to fresh NGM plates seeded with *E. coli OP50* or *L. reuteri* A21041 at 20 °C, and there were at least 30 worms for each group. Head swing rate was measured by counting head swings per 30s of each worm moving in M9 buffer and body bend rate was assayed on a fresh NGM plate on day 5, day 10, and day 15. The time when the L4 stage nematodes were picked onto the plates was recorded as day 0. This experiment was repeated three times.

### Oil red O staining

Oil red O staining assay was performed as described by Wang et al. (28). For this, 100 L4 stage worms were transferred to fresh NGM plates covered by OP50 or A21041 at 20 °C and the time when the L4 stage nematodes were picked onto the plates was recorded as day 0. In this experiment, we set two time points, namely the 5th day and the 7th day. On days 5 and 7, worms were collected in a tube containing M9 buffer and washed three times, then resuspended in 4% paraformaldehyde. A mixture of isopropanol and triton X-100 was used to re-suspend the worms after discarding paraformaldehyde. After 15 min, we removed the isopropanol supernatant and added 1 mL Oil red O to tubes to stain the worms for 2 h. Images of worms were obtained using the Leica Microsystems CMS GmbH microscope and analyzed using ImageJ software. This experiment was repeated three times.

### Stress-resistance assays

The stress-resistance assays were based on Xiao’s study, with small modifications (29). For the thermal shock assay, about 35 L4 stage worms were transferred to fresh NGM plates covered by OP50 or A21041 at 20 °C in the first 4 days. On day 5, temperatures were increased to 37 °C and dead worms were counted per hour until all worms died. For H_2_O_2_-induced oxidative stress assay, 35 age-synchronized young adult worms were treated with A21041 and *E. coli* OP50 at 20 °C. On day 5, worms were transferred to fresh NGM plates containing H_2_O_2_ at a final concentration of 3.75 mM. Dead worms were counted per hour until they all died. The time when the L4 stage nematodes were picked onto the plates was recorded as day 0. Three independent stress-resistance assays were performed.

### SOD assays

The SOD activity was assessed by the SOD detection kit (Jiancheng, Nanjing, China). For worm models, 200 L4 stage worms were routinely fed with OP50 or A21041 and the day the worms were picked onto plates was recorded as day 0. On day 5, worms were collected and washed with M9 buffer and then tested with the kit mentioned above. Protein level was normalized using the standard 1 Abs = 1 mg/mL. A microplate reader (Tecan Infinite® 200 PRO, Wuhan, China) was used to measure the enzymes level and the experiment was repeated three times.

### RNA sequencing

RNA sequencing was performed twice through Ilumina NovaSeq 6000 (Novogene, Beijing, China) to investigate the signaling pathways of *C. elegans* with the A21041treatment. About 2000 L4 worms were fed OP50 or A21041 and collected at day 5 for RNA-seq. Fastp (v0.21.0) was used to prune the RNA-seq data and filter the low-quality sequences (parameter-q 20, with the remaining being the default parameters), and hisat2 (v2.1.0) was used to align the filtered RNA-seq reads to WBcel235. The mapped reads were quantified by htseq (v0.11.2) and the genes with low expression were filtered; the final reads were normalized by TPM (Transcripts Per Million) and FPKM (Fragments Per Kilobase Per Million). The analysis of gene expression differences between different groups was performed using the edgeR package (v3.40.2) of R language, and the differentially expressed genes were screened with *p* < 0.05 and Log_2_ FC (fold change) > 1.5 or <−1.5. GO and KEGG enrichment analyses were performed for differentially expressed genes using the clusterProfiler package (v4.6.2) in R language.

A quantitative real-time PCR (qPCR) assay was performed to assess genes expression level. For the worm model, about 1,000 L4 stage worms were transferred to fresh NGM plates grown with OP50 or A21041. Worms were collected for all qPCR assays in this study on day 5. The day the nematodes were picked onto plates was recorded as day 0. Worms from the two groups were collected and washed twice with M9 buffer. Total RNA was extracted using a total RNA extraction kit (Promega, Shanghai, China) and cDNA was obtained using a reverse transcription kit (Tiangen, Beijing, China). Primers were designed through PrimerQuest™ Tool. Quantitative real-time PCR analysis was performed on a QuantStudi® 3 Real-Time PCR Instrument (Applied Biosystems, Massachusetts, USA) and beta-actin, a house-keeping gene, was used as an endogenous reference gene. The relative expression levels of target genes were calculated using the comparative CT (2^−ΔΔCT^) method.

### Metabolomics assays

For the untargeted metabolomics assay, approximately 3000 L4 stage worms were transferred to fresh NGM plates grown with OP50 or A21041 on day 5. The bacteria of OP50 and A21041 were collected from the plates and washed twice with M9 buffer. After removing the supernatant, the bacteria were frozen at once using liquid nitrogen. Metabolomics analysis was performed twice based on liquid chromatography-mass spectrometry (LC–MS) (Novogene, Beijing, China). Most of the metabolites were identified according to mzCloud, mzVault, and MassList databases. Public databases such as Kyoto Encyclopedia of Genes and Genomes (KEGG, https://www.genome.jp/kegg/pathway.html), Human Metabolome Database (HMDB, https://hmdb.ca/metabolites), and Lipid metabolites and pathways strategy (LIPID MAPS, https://www.lipidmaps.org/resources/pathways) were used to elucidate the functions and pathways of these metabolites.

### Statistical analysis

The data were analyzed using GraphPad (v9.5.0) based on the *T* test, Kaplan–Meier analysis, Log-rank (Mantel–Cox) test, and Tukey–Kramer multiple-comparison test at a *p* < 0.05 or *p* < 0.01 level of significance.

## Results

### *Limosilactobacillus reuteri* A21041 increased the lifespan and motility of *Caenorhabditis elegans*

**Figure 1:**
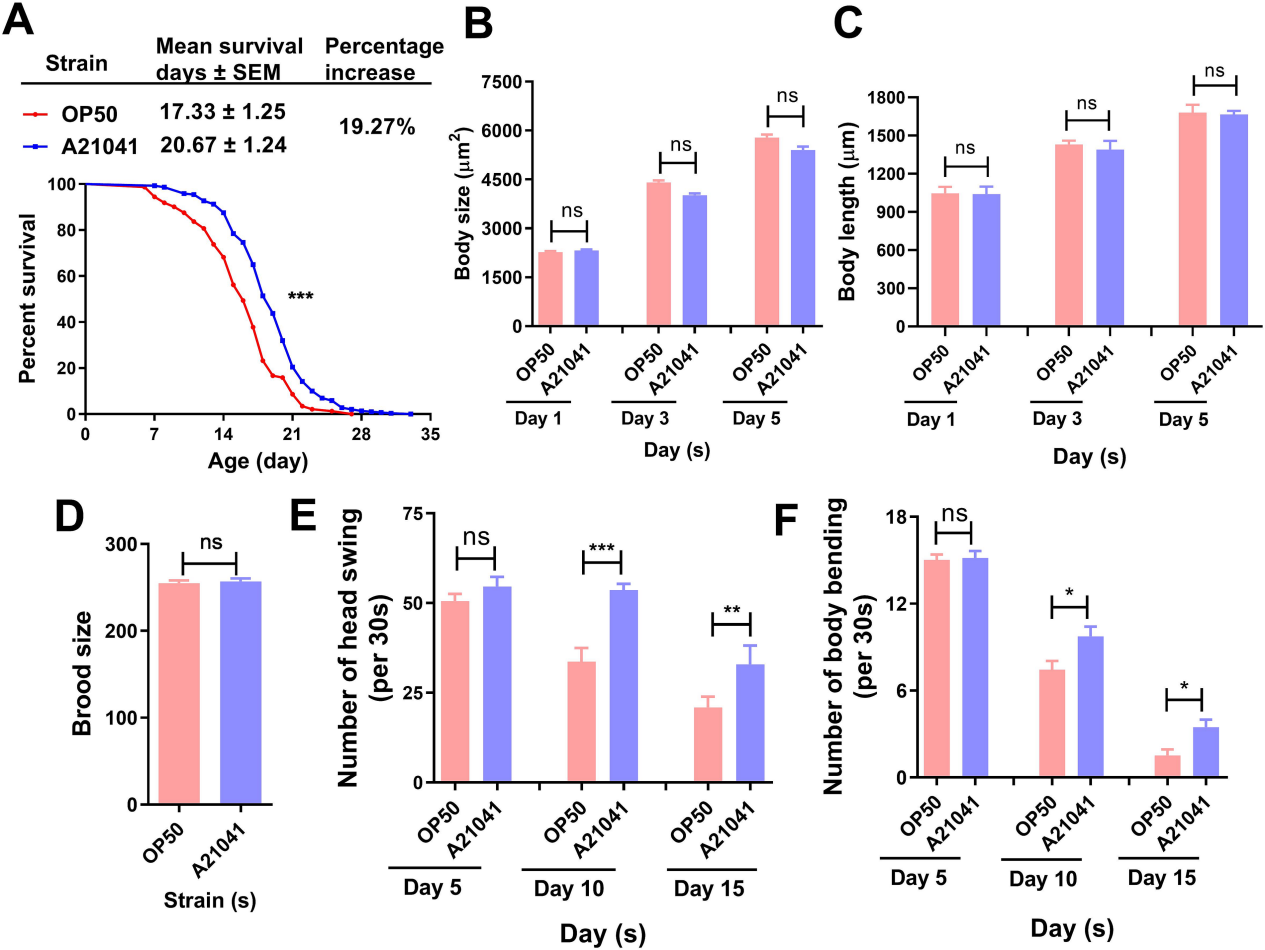
*Lactobacillus reuteri* A21041 promotes the lifespan and motility of *Caenorhabditis elegans* but does not affect body size or fertility. **(A)** A comparison of A21041 and the control group *Escherichia coli* OP50 on survival rate of *Caenorhabditis elegans*, *n* = 200. **(B)** The effect of A21041 on body size compared with *Escherichia coli* OP50 *n* = 20. **(C)** Body length measurement of nematodes fed with OP50 or A21041, *n* = 20. **(D)** Total number of eggs laid, *n* = 10. **(E)** The effects of A21041 feeding on motility of head swing on days 5, 10, and 15 respectively, *n* = 30. **(F)** The effects of A21041 feeding on motility of body bending on days 5, 10, and 15 respectively, *n* = 30.

We first investigated whether A21041 could affect the lifespan of *C. elegans*. As shown in Figure 1A, feeding the worms with A21041 significantly prolonged the lifespan of *C. elegans* by 19.27% (medium survival rate analysis) compared with the OP50 control group. In addition, neither the body size and length (Figures 1B,C) nor the fertility (Figure 1D) of the worms were affected upon A2104 feeding. To evaluate the effect of A21041 on the general health of the worms, motility assays were performed, including the frequency of head swings (Figure 1E) and body bending (Figure 1F). Compared to the control group, A21041 feeding significantly improved the frequency of head swings of the worms by 59.23% on day 10 and 57.66% on day 15, and body bending increased by 30.85% on day 10 and 127.5% on day 15. Motility tests showed that A21041 feeding obviously promoted the locomotive function of adult and aged worms. These results together indicate that A21041 could extend the lifespan and improve the general health of the worms.

### A21041 reduced fat accumulation in *Caenorhabditis elegans*

**Figure 2:**
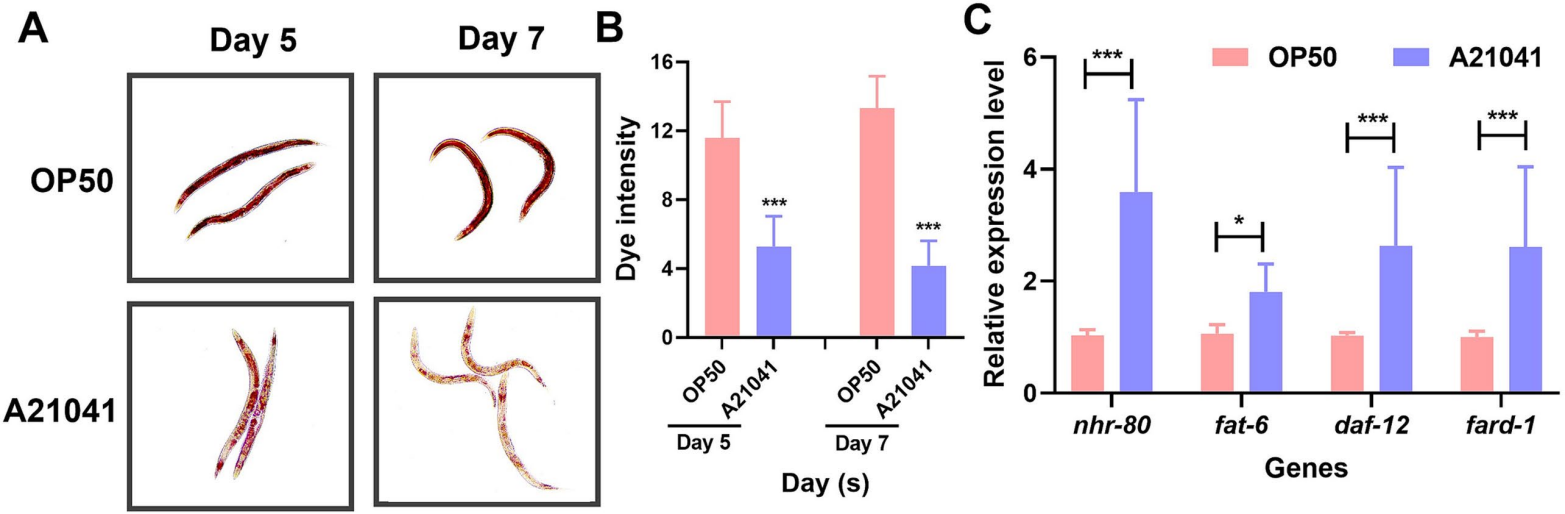
A21041 decreases the fat storage of *Caenorhabditis elegans*. **(A)** The microscope images compare the lipid storage of *Caenorhabditis elegans* with the treatment of Oil red O staining between *Escherichia coli* OP50 and A21041 on day 5 (*n* = 35) and day 7 (*n* = 35). **(B)** Dye intensity statistical analysis through Image J. **(C)** Relative expression levels of fat-regulation genes *nhr-80*, *fat-6*, *daf-12*, and *fard-1* in *Caenorhabditis elegans* after feeding with A21041, *n* = 1,000.

It is known that lifespan prolongation is related to lipid metabolism in *C. elegans* (30), thus we examined whether A21041 reduced lipid storage in the worms. Oil Red O, a soluble dye in lipids, was used to quantify fat distribution. A significant decrease in dye intensity was observed in the A21041-treated worms (Figure 2A). Compared with the control group, fat accumulation of the A21041 group decreased by 54.47 and 68.77% on days 5 and 7, respectively (Figure 2B). Consistent with the observation above, the gene expression levels of *nhr-80*, *fat-6*, *daf-12*, and *fard-1*, which are involved in the fatty acid desaturation pathway, was significantly increased in worms treated with A21041 (Figure 2C). These results together indicate that A21041 treatment could reduce fat accumulation in the worms.

### Effects of A21041 on stress resistance in *Caenorhabditis elegans*

**Figure 3:**
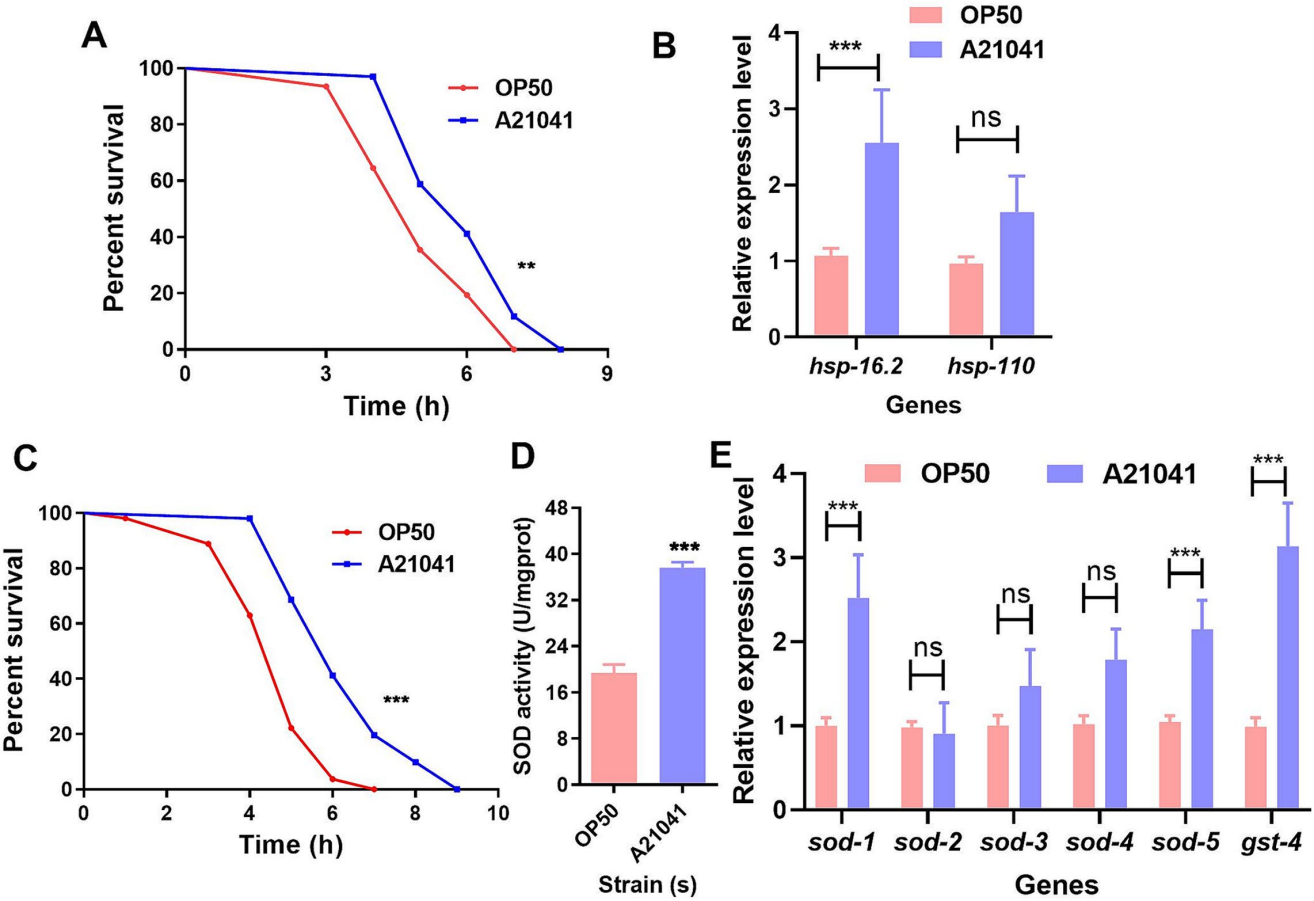
A21041 increases stress resistance in worms. **(A)** A21041 treatment significantly improves heat resistance in *Caenorhabditis elegans*, *n* = 35. **(B)** Relative expression level of genes encoding heat shock proteins in *Caenorhabditis elegans* after feeding with A21041. **(C)** A21041-fed worms improve their oxidative stress response, *n* = 35. **(D)** SOD activity in nematodes after the treatment with *Escherichia coli* OP50 or A21041, *n* = 200. **(E)** Relative expression level of genes associated with stress resistance in *Caenorhabditis elegans*, *n* = 1,000.

Environmental stress can influence *C. elegans’* lifespan (31), thus we performed stress-resistance assays to test whether probiotic A21041 feeding could help the worms to survive longer under extreme environmental conditions such as high temperature and oxidative stress. As shown in Figure 3A, A21041 treatment improved the lifespan of worms by 20% in a thermal shock assay. Next, qPCR assays were performed to investigate whether genes encoding classical heat shock proteins (HSP) would be affected. Indeed, we found that the *hsp-16.2* mRNA level was up-regulated by 155.45% and *hsp-110* expression level was also increased even though it did not show statistical significance (Figure 3B). In addition, feeding with A21041 increased the survival of the worms by 40% in an oxidative assay via medium survival rate analysis (Figure 3C). Furthermore, we tested the SOD activity in the worms upon A21041 treatment. Feeding with A21041 increased SOD activity in *C. elegans* by 95.53% (Figure 3D). There are five SOD genes encoding cytosolic SOD enzymes: *sod-1*, *sod-5*, mitochondrial SOD enzymes *sod-2* and *sod-3*, and extracellular SOD enzyme *sod-4* (31). Based on these five enzymes, we found that *sod-1* and *sod-5* were upregulated significantly whereas *sod-3* and *sod-4* expression also increased although with no statistical significance by qPCR analysis (Figure 3E), indicating that the worms might survive longer under an oxidative environment through regulating SOD activity. Additionally, the gene *gst-4* was also associated with stress resistance (32) and expression level of this gene was up-regulated significantly after A21041 treatment (Figure 3E) compared with the control.

### A21041 extended lifespan through the insulin-like pathway

**Figure 4:**
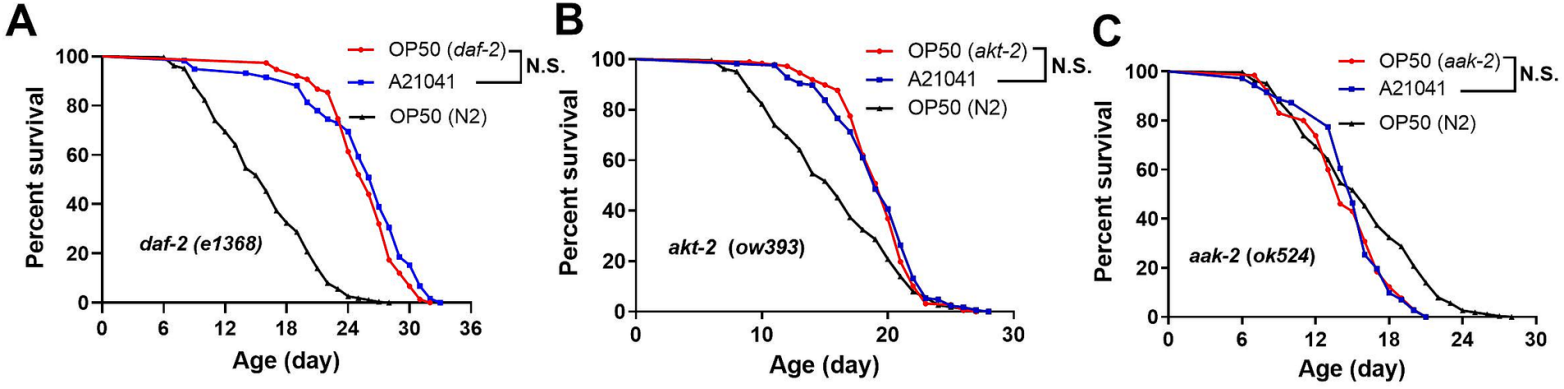
Survival curves of parts of mutants in longevity-regulating pathways with *Escherichia coli* OP50 or A21041 feeding. **(A)** *daf-2* (*e1368*), *n* = 200. **(B)** *akt-2* (*ow393*), *n* = 200. **(C)** *aak-2* (*ok524*), *n* = 200.

To further explore which genes in the worms may participate in the A21041-induced longevity, lifespan assays were performed in loss-of-function mutants in the insulin-like pathway based on Zhang’s et al. (33) study with small modifications. As shown in Figure 4A, A21041 feeding failed to prolong the lifespan of the worms with a *daf-2* mutation, which was a key component of the insulin/IGF-1 signaling (IIS) pathway and mediated the signaling of worms entering the reproduction period or staying in the long-lived dauer diapause stage (34). Reducing the expression of *daf-2* could extend the lifespan (33). AKT is a downstream target of *daf-2* in the IIS signaling (35). As shown in Figure 4A, the lifespan extension effect was abolished in the *akt-2* mutant (Figure 4B). These results indicate that A21041 promoted the longevity of the worms via the IIS pathway. Moreover, feeding with A21041 did not affect the lifespan of mutant *aak-2* (Figure 4C). As previously reported, dietary restriction (DR) can delay aging in many species, but the worms under DR condition had smaller body sizes than those cultivated with *E. coli* OP50 (36, 37). Loss-of-function mutants *eat-2* and *aak-2* are representatives of DR pathway and AMPK cascade, respectively, (38). As shown in Figures 1B,C, there were no significant changes between the body size of two groups, suggesting that A21041-induced longevity was independent of DR signaling and might depend on AMPK pathway.

In addition, A21041 significantly extended the lifespan of mutants *eat-1*, *skn-1*, *age-1*, *daf-16*, *daf-18, daf-28, pmk-1, sek-1*, and *nsy-1* in wild-type N2 (Supplementary Figures 1A-I), which suggested that these target genes were irrelevant to the longevity induced by A21041.

### Mechanism of feeding A21041 promoted the lifespan of *Caenorhabditis elegans*

**Figure 5:**
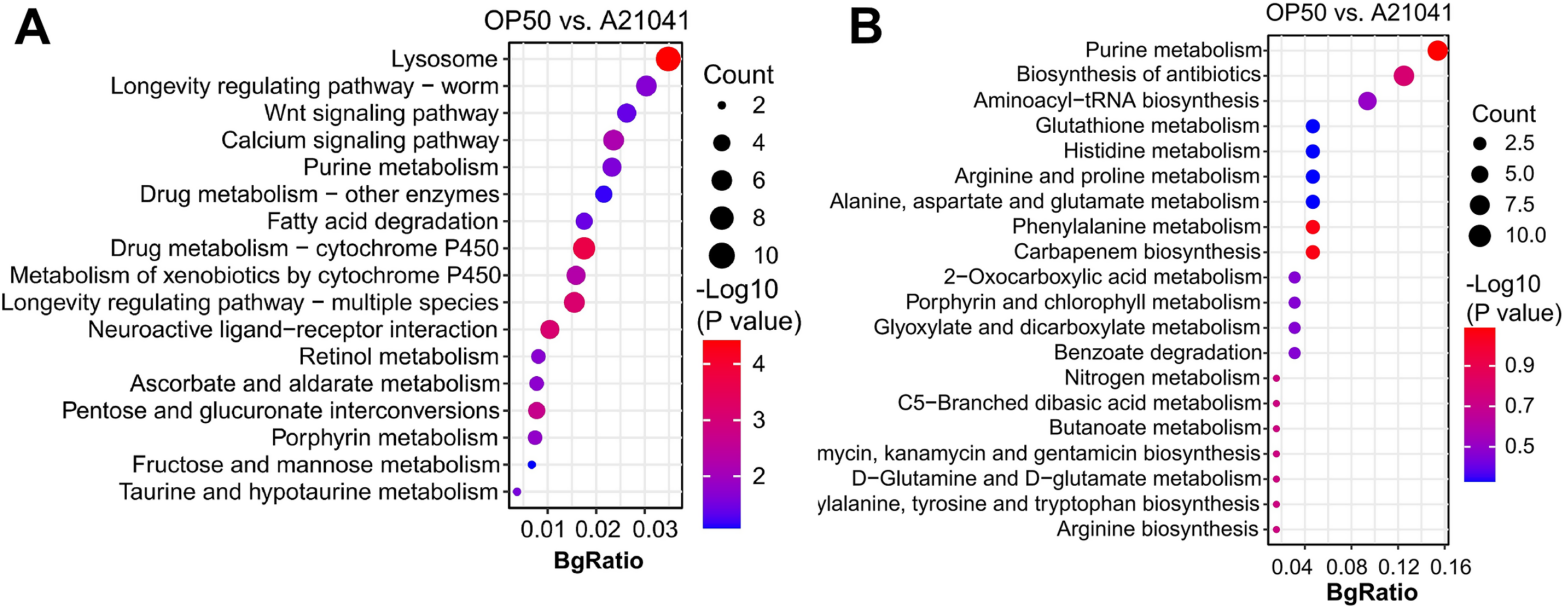
Transcriptomics and metabolomics analysis in the worm model. **(A)** Transcriptomics analysis reveals that A21041 extends the lifespan of *Caenorhabditis elegans* by regulating lysosome, longevity-regulating pathways, fatty acid degradation signaling, and drug metabolism cascades, *n* = 2,000. **(B)** Metabolomics analysis illustrates that A21041 extends the longevity of worms by affecting purine metabolism, biosynthesis of antibiotics, and phenylalanine metabolism pathways, *n* = 3,000.

**Figure 6:**
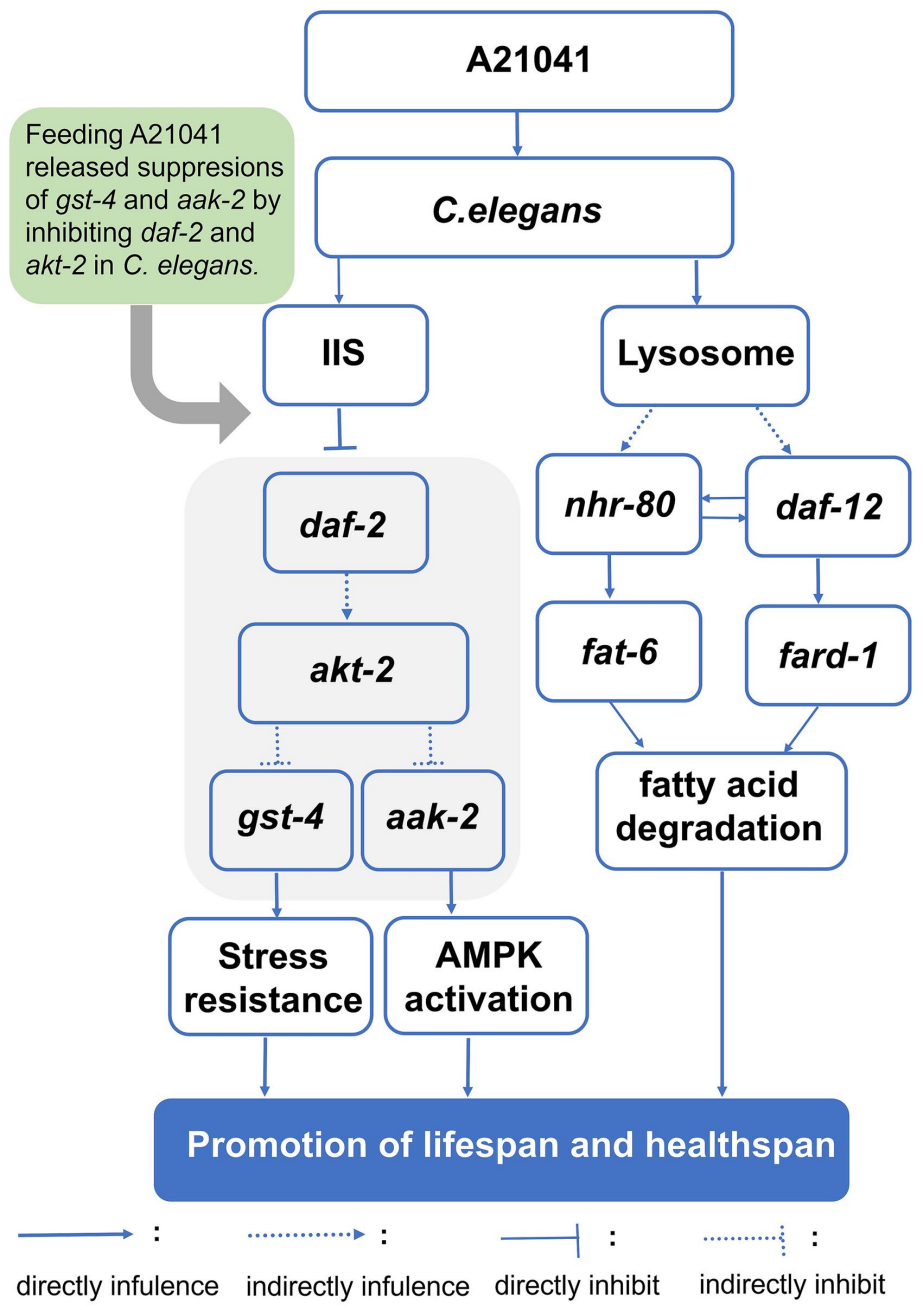
The schematic diagram of the proposed mechanism of A21041-induced longevity and health-promoting effects in *Caenorhabditis elegans*.

RNA sequencing analysis was performed to examine how A21041 treatment affected the transcriptome profile of worms. RNA sequencing analysis showed that A21041-induced longevity involved lysosome, longevity regulating pathways, and fatty acid degradation signaling. (Figure 5A). Transcriptome analysis showed that the expression levels of 332 genes increased whereas down-regulation occurred in 15 genes after feeding A21041 (Supplementary Figure 2). qPCR assays were performed to identify the accuracy rate of RNA sequencing results; 100% of genes were regulated accordingly as transcriptome analysis (Supplementary Figure 3), which suggested that RNA sequencing analysis was relatively reliable. Most of the genes used in this study showed the same trends as in the RNA-seq data, although there was no obvious significance for some genes between A21041 and OP50 (data not shown). Through transcriptome KEGG enrichment analysis, we found that *gst-4*, which was confirmed to enhance resistance and extend longevity (39), was significantly up-regulated in the A21041-fed group (Supplementary Figure 4), which was consistent with our experimental results in Figure 3G. Then we found that *atf-4* was significantly up-regulated in A21041-fed worms (Supplementary Figure 4). Studies have shown that *atf-4* promotes lifespan through improving hydrogen sulfide (H_2_S) production with the CTH-2 enzyme, and the ATF-4/CTH-2/H_2_S pathway extended the lifespan of *C. elegans* by inhibiting the mechanistic target of rapamycin complex 1 (mTORC1) (40). Surprisingly, gene *cth-2*, which encodes the CTH-2 enzyme, was also upregulated obviously through RNA-seq analysis which suggested that A21041-mediated longevity of *C. elegans* might depend on ATF-4/CTH-2/H_2_S pathway. In addition, qPCR analysis (Figure 2C) showed that feeding with A21041 significantly upregulated the gene expression of *nrh-80* and *daf-12*, which are nuclear transcription factors from lysosomal signaling pathways that regulate lifespan prolongation, in *C. elegans* (Figure 6), and *fat-6* and *fard-1* were downstream genes of *nrh-80* and *daf-12, respectively,* which were associated with fatty acid degradation (41). Consistent with our finding, Yu et al. (30) showed that ginsenoside reduced lipofuscin accumulation via *nrh-80* up-regulation.

We also performed metabolomics analysis to identify significantly regulated metabolites related to the mechanisms underlying A21041 and OP50. As shown in Supplementary Figure 5, 81 metabolites, which were classified into organic acids and derivatives, lipids, and lipid-like molecules, presented obvious regulation between A21041 and OP50 groups. These metabolites were mainly enriched in purine metabolism, biosynthesis of antibiotics, and phenylalanine metabolism pathways (Figure 5B). Among these metabolites, the expression levels of guanosine, spectinomycin, deoxycholic acid, and syringic acid in the A21041-fed group was higher than the OP50 group (Supplementary Table 1). Arachidonic acid, linoleic acid, and proinflammatory factors exhibited lower expression in A21041-fed worms than OP50 group (Supplementary Table 1) (42). Additionally, six metabolites were classified into indoles and derivatives (Supplementary Table 1), four of which were significantly up-regulated, including 5-Hydroxytryptophol, 5-Hydroxyindole-3-acetic acid, and 5-Hydroxytryptophan.

## Discussion

Probiotics provide benefits to their host by regulating the immune system, resisting oxidative and environmental stress, and improving gut micro-ecological balance (43, 44). A large number of probiotics have been found that can improve the lifespan and healthspan of many kinds of model animals such as yeasts, nematodes, fruit flies, mice, and monkeys (45). In this study, we defined healthy longevity as living longer naturally, being more physically capable, and having a stronger stress response to environmental challenges. We set up a series method using *C. elegans* based on reported research to screen different functional strains from the gut microbes of centenarians. Among these strains, we surprisedly found that *L. reuteri* A21041 could promote lifespan in *C. elegans*, improve motility significantly in adult and aged nematodes, decrease lipid storage by more than 50% in adult worms, increase heat resistance and antioxidant ability significantly, and not influence body size and fertility.

This study showed that *L. reuteri* A21041 was able to extend the lifespan of *C. elegans* by 19.27% and promote the healthspan of this model organism through motility assays (Figure 1). Similarly, Lee et al. (46) found that *L. reuteri* promoted the lifespan of *Drosophila Melanogaster* by 9% via regulating the IIS pathway. Sciandrone et al. (47) also conducted lifespan assays using *C. elegans* to evaluate the effect of *L. reuteri* PBS072, but the median lifespan of worms did not show significance. Fat accumulation can lower autophagy ability and hinder the recycling of cell components (48). Several fat metabolic pathways identified in *C. elegans* have also been identified in mammals, thus *C. elegans* is an excellent model for lipid storage assays (49). A21041 feeding decreased lipid storage by 54.47 and 68.77% on day 5 and day 7, respectively, via upregulated genes expression of *nhr-80*, *daf-12*, and *fard-1* etc. (Figure 2), which indicated that A21041 might reduce fat storage of *C. elegans* by lysosome pathway (Figure 6). *L. reuteri* A21041 increased the ability of worms against hyperthermia significantly compared with *E. coli* OP50 group (Figure 3A). Heat stress activates the heat shock response (HSR), which was required for thermos-resistance and longevity (50). Accordingly, we found that classical heat response proteins, such as *hsp-16.2*, were up-regulated significantly through qPCR assays (Figure 3B). For *C. elegans*, HSR was the main driver behind maintaining protein homeostasis and protecting proteomes of cytosol under environmental stress and *hsp-16.2* was an important gene in HSR (51–53). Therefore, A21041 feeding improved the heat resistance of *C. elegans*. Additionally, A21041 also significantly enhanced the oxidation stress resistance of worms in the presence of H₂O₂ (Figure 3C) via increasing SOD activity (Figure 3D). qPCR analysis revealed that genes *sod-1* and *sod-5* were obviously upregulated, which indicated that A21041 enhanced SOD activity by cytosolic SOD enzymes mainly (Figure 3E). Yanase et al. (54) found that *sod-1* helped remove superoxide radicals in host cytoplasm and mitochondria while *sod-5* would compensate by being expressed when *sod-1* was missing. Moreover, it is well-known that *gst-4* upregulation is closely related to antioxidant ability increase and lifespan extension (32, 39) and this gene in the A21041-fed group was significantly up-regulated compared with the OP50 group both in qPCR or RNA-seq analysis, which indicated that *gst-4* also participated in A21041-mediated antioxidant effect and longevity (Figure 3E, Supplementary Figure 4). Similar to our results, Lu et al. (55) illustrated that *Latilactobacillus curvatus* FFZZH5L increased the antioxidant capacity of *C. elegans* by up-regulating *gst-4*. Pohl et al. (56) revealed that *gst-4* was a vitally important target for drug screening of neurodegenerative diseases on account of its strong antioxidant properties.

To further explore the longevity mechanism induced by A21041, we selected mutants from different pathways for lifespan experiments. As shown in Figure 4, Supplementary Figure 1, feeding with A21041 failed to extend the lifespan of mutants *daf-2*, *akt-2*, and *aak-2*, which indicates that A21041-induced changes may be regulated by these genes, while A21041 significantly extended the lifespan of mutants *eat-1*, *skn-1*, *age-1*, *daf-16*, *daf-18, daf-28, pmk-1, sek-*,*1* and *nsy-1* as in wild-type N2. Therefore, A21041-induced longevity may depend on IIS signaling pathways based on the lifespan assays of mutants (Figure 6). *daf-2* is a key receptor homolog upstream of IIS signaling pathways and it plays an important role in regulating longevity, motility, and stress resistance (33, 57–59). Gene *akt-2* acts downstream of *daf-2* and upstream of *skn-1* (60). Meanwhile, the p38 MAPK pathway, namely TIR-1–NSY-1–SEK-1–PMK-1, is well-known to be involved in immunity against infections (61). For instance, *Lactobacillus curvatus BGMK2-41* stimulated the immune response against *Pseudomonas aeruginosa* and *Staphylococcus aureus* by regulating p38 MAPK cascade reaction (62). SKN-1, an oxidative stress response transcription factor, is also the downstream target of *pmk-1* (61). Feeding A21041 did not require *nsy-1*, *sek-1*, *pmk-1*, or *skn-1* to prolong worm’s lifespan which implies that A21041 did not mainly depend on p38 MAPK cascade. T *gst-4* is the downstream gene of *skn-1* (32, 39), but A21041-induced antioxidant ability depended on *gst-4* rather than *skn-1*, which indicated the up-regulation of *gst-4* might be mediated through another pathway. Additionally, lifespan promotion of *C. elegans* depended on *aak-2* rather than *eat-1*, suggesting that A21041-induced longevity might be independent of the DR signaling pathway and dependent on the AMPK pathway. It is well-known that AMPK activation can slow down ageing (63). Interestingly, *daf-2*-caused longevity highly depended on *aak-2* while *eat-1*-induced lifespan prolongation was independent of *aak-2* (64) which was in line with our results. In the IIS pathway, *akt-2* and *aak-2* are in different branches and act as opposing regulatory signals (39). When *akt-2* is inhibited, *aak-2* activates and helps maintain energy balance by activating the AMPK pathway (39). Thus, A21041 might activate *gst-4* and *aak-2* by suppressing *daf-2* and *akt-2*, as shown in Figure 6.

Moreover, transcriptome sequencing and metabolome analysis of *C. elegans* showed that differently expressed genes and metabolites were associated with longevity regulation, fatty acid degradation, and hyperthermia stress and oxidative stress resistance based on the KEGG database, which is consistent with the main experimental results in this study (Figures 5, 6). Except for the significant up-regulation of *gst-4*, we still found that *atf-4* and *cth-2*, which were highly associated with lifespan promotion of *C. elegans*, presented significant up-regulation in A21041 treatment group which indicated that ATF-4/CTH-2/H2S pathway participated in the lifespan-extending effect mediated by A21041 (Supplementary Figure 4). Moreover, the different expressions of metabolites illustrated that A21041 had strong antioxidant ability and was able to help the host improve their healthspan by promoting the anti-oxidation function (Figure 5B) which was consistent with our results in Figure 3. da Silveira et al. (65) found that guanosine prevents excitotoxicity in *C. elegans* (66) and spectinomycin was a kind of broad-spectrum antibiotic which might protect *C. elegans* from pathogenic bacteria (67). Deoxycholic acid was widely used in fat reduction, which might be the reason of less lipid storage in A21041-fed group (65). Many investigations verified that syringic acid was effective in alleviating oxidative stress and inflammation (68). Ferah Okkay et al. (69) also proposed that syringic acid increased SOD activity. Additionally, many reports have revealed the effects of indole metabolites in the development of tumors, chemotherapy and immunotherapy (70). 5-Hydroxytryptophan specifically was reported to impact on the regulation of sleep, depression, anxiety, aggression, sexual behavior, and pain sensation (71) and was up-regulated significantly in A21041-fed group compared with OP50 group.

Finally, there were some limitations to this study. Firstly, we did not further test A21041’s effects on lifespan extension, fat reduction, or antioxidant benefits in higher animals such as mice or monkeys. Our mutant experiments also did not fully cover the lifespan extension pathway targets. We will take a closer look at the mechanism of A21041 in our follow-up research. In summary, *L. reuteri* A21041 treatment could significantly extend the lifespan and healthspan of *C. elegans* by regulating IIS signaling pathways and the lysosome signaling pathway. Additionally, A21041 has been shown to have a strong survival ability in artificial gastrointestinal fluids and bile salts based on our previous studies (23, 24). Therefore, A21041 could be a promising probiotic in dietary supplements.

## Data Availability

The data analyzed and/or generated in this study can be found in the NCBI (https://www.ncbi.nlm.nih.gov/) under BioProject accession PRJNA1468957.
