# Supplementary material for: Effects and mechanisms of lifespan extension and healthspan promotion induced by Limosilactobacillus reuteri (Lactobacillus reuteri) A21041
Source: Front Nutr. 2026 Jul 10;13:1852156. doi: 10.3389/fnut.2026.1852156 (PMC13396012; doi:10.3389/fnut.2026.1852156)
Supplement: Supplementary file 1 [file Supplementary_file_1.pdf]

Effects and Mechanisms of Lifespan Extension and Healthspan Promotion Induced by *Limosilactobacillus reuteri* (*Lactobacillus reuteri*) A21041

Lili Meng[1,2], Lianfei Huang[1,2], Enqiu Lu[1,2],, Zhihui Chai[3], Zhilong Lu[1,2,3], Shifu Pang[1,2], Xinli Wei[1,2], Manping Chen[1,2,4], Guilong Xiao[1,2,4], Yanjin Lin[1,2,4] and Weifei Luo[1,2,3*]

1. AIage Longevity Science Corporation Ltd, Shenzhen, Guangdong, China.
2. Guangxi Key Laboratory of Longevity Science and Technology, Guangxi AIage Longevity Science Corporation Ltd, Nanning, Guangxi, China.
3. National Key Laboratory of Non-food Biomass Energy Technology, Institute of Biological Science and Technology, Guangxi Academy of Sciences, Nanning, Guangxi, China.
4. Sichuan Alage Life Science Corporation Ltd, Dazhou, Sichuan, China.

*Correspondence to Weifei Luo, Luoweifei@gxas.cn.

Running title: *L. reuteri* A21041 extended lifespan and promoted healthspan.

Supplementary materials:

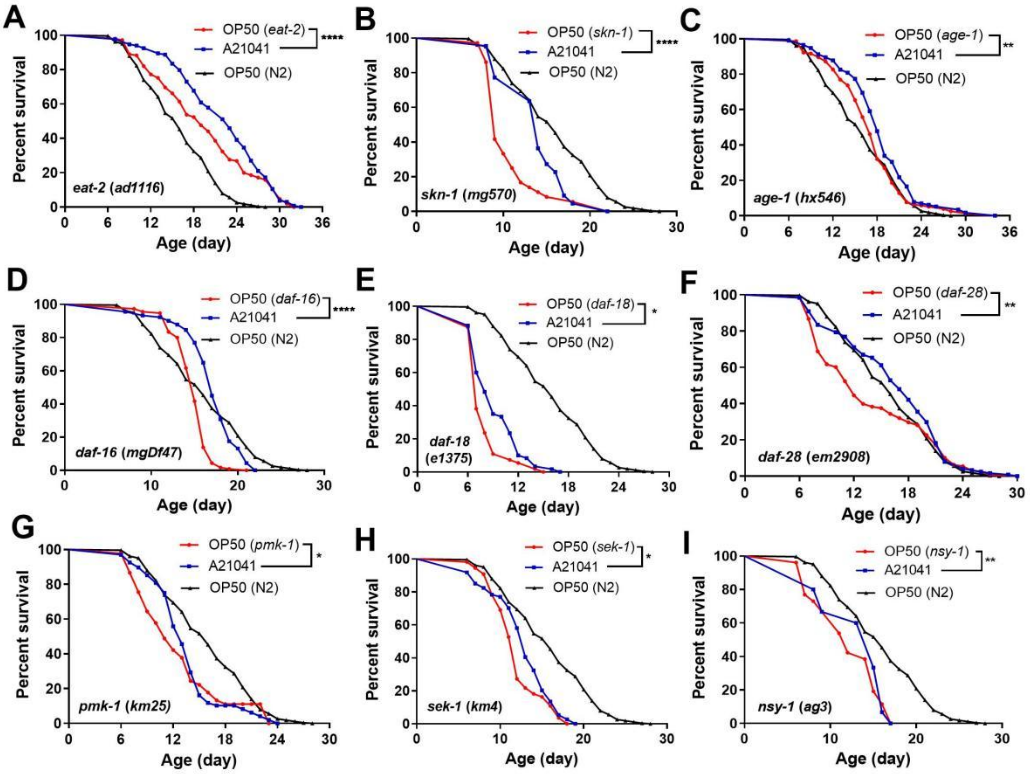

FIGURE 1 Survival curves of mutants involved in longevity regulation of *C. elegans with* feeding A21041 or *E. coli* OP50. **(A)** *eat-2* (*ad1116*), n=200. **(B)** *skn-1* (*mg570*), n=200. **(C)** *age-1* (*hx546*), n=200. **(D)** *daf-16* (*mgDF47*), n=200. **(E)** *daf-18* (*e1375*), n=200. **(F)** *daf-28* (*em2980*), n=200. (G) *pmk-1* (*km25*), n=200. (H) *sek-1* (*km4*), n=200. (I) *nsy-1* (*ag3*), n=200.

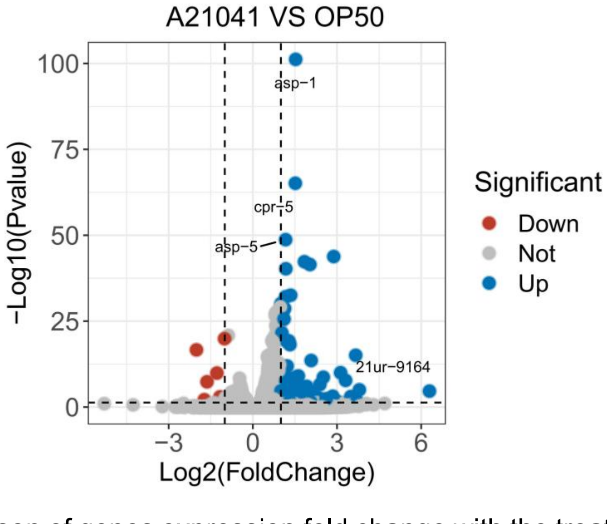

FIGURE 2 Comparison of genes expression fold change with the treatment of *E. coli* OP50 or A21041 in *C. elegans* via RNA sequencing.

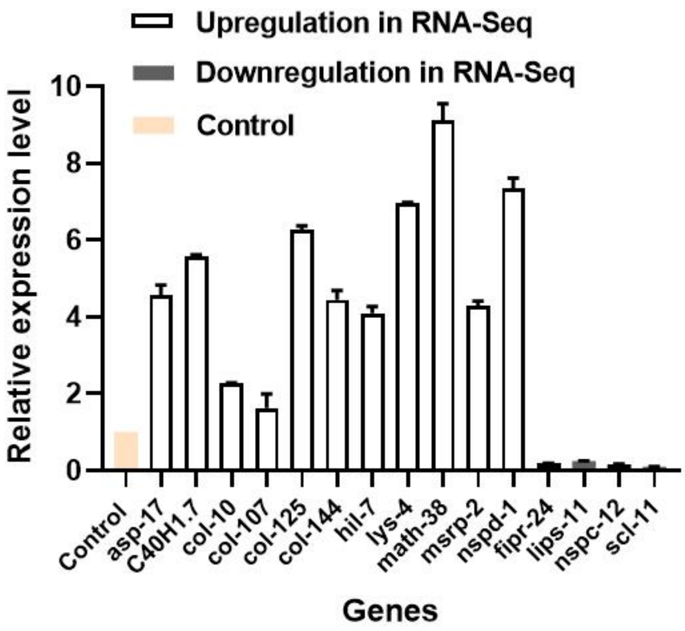

FIGURE 3 qPCR analysis inhibits that accuracy rate of transcriptomics analysis is 100%.

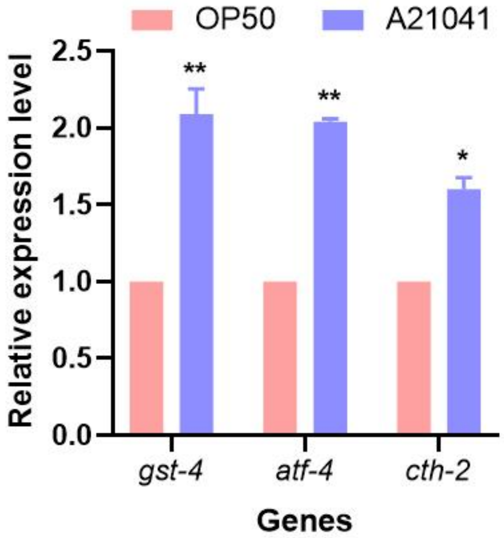

FIGURE 4 The relative expression level of genes of *gst-4*, *atf-4* and *cth-2* through RNA-seq analysis.

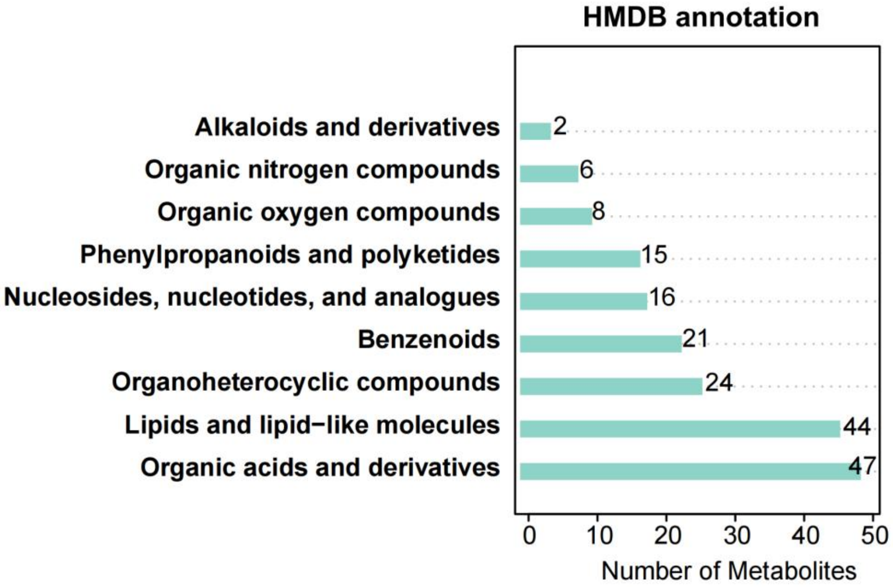

FIGURE 5 Differentially expressed metabolites of A21041 and *E. coli* OP50 in HMDB.

Table 1 Part of important metabolites of metabolome analysis.

Name	Class	log2 FC
Guanosine	Purine nucleosides	1.39
Spectinomycin	Dioxanes	0.79
Deoxycholic acid	Steroids and steroid derivatives	0.88
Syringic acid	Benzene and substituted derivatives	3.82
Arachidonic acid	Fatty Acyls	-1.57
Linoleic Acid	Fatty Acyls	-1.85
5-Hydroxytryptophol	Indoles and derivatives	4.96
5-Hydroxyindole-3-acetic acid	Indoles and derivatives	2.80
5-Hydroxytryptophan	Indoles and derivatives	1.93
JWH 250 N-pentanoic acid metabolite	Indoles and derivatives	3.00
trans-3-Indoleacrylic acid	Indoles and derivatives	1.40
5-Hydroxyindole	Indoles and derivatives	-0.70
